# Comment on: Anti-Oxidative Stress Activity of Stachys lavandulifolia Aqueous Extract in Humans

**Published:** 2013-08-24

**Authors:** Azar Baradaran, Hamid Nasri, Mahmoud Rafieian-Kopaei

**Affiliations:** 1 Department of Pathology, Isfahan University of Medical Sciences, Isfahan, Iran; 2 Department of Nephrology, Division of Nephropathology, Isfahan University of Medical Sciences, Isfahan, Iran; 3 Medical Plants Research Center, Shahrekord University of Medical Sciences, Shahrekord, Iran

**Keywords:** Stachys lavandulifolia, Toxicity, Nephrotoxicity, Antioxidant

## Abstract

A recent article by Rahzani et al. (1) published in the esteemed Cell Journal reported the antioxidative stress activity of Stachys lavandulifolia aqueous extract in humans and suggested its consumption as a supplement in the management of diseases related to oxidative stress. We would like to emphasize some of the limitations regarding antioxidant supplementation, in general, and Stachys lavandulifolia, in particular.

It has been established that oxidative stress is involved in the development of a wide variety of chronic and degenerative diseases such as cancer, Parkinson and Alzheimer’s (2-5). Antioxidants are also effective in the prevention or reduction of adverse effects related to medication usage (5-10). However, they may potentially have deleterious effects. A major concern of antioxidant supplementation is their harmful effect on reactive oxygen species (ROS) production (prooxidant action), particularly when precise modulation of ROS levels are necessary for normal cell function (4-10). In fact, it has been reported that antioxidants may exhibit pro-oxidant activity under specific conditions. Of particular importance are redox conditions the dosage and the presence of free transition metals at cellular sites. For example, the antioxidant vitamin C in the presence of ferric iron may act as a potent mediator of lipid peroxidation. It has been suggested that β-carotene sometimes acts as a pro-oxidant in the lungs of smokers and similarly vitamin C may increase DNA damage in humans (11, 12). Therefore, it is necessary to take into account the bioavailability and differential activities of antioxidant compounds before their administration.

Other than general considerations for antioxidant consumption, the aspects of each particular antioxidant should also be considered (3, 13, 14). Recently, in a preclinical study we reported the renal toxicity of hydroalcoholic extract of Stachys lavandulifolia Vahl in Wistar rats (15). In this experimental study we randomly assigned 100 male Wistar rats to five equal groups, one control and four experimental. Animals received intraperitoneal injections of saline or Stachys lavandulifolia extract (50, 100, 150, 200 mg/kg) for one month after which blood samples were collected from half of the animals from each group. Other animals received no injections for one additional month, then blood samples were obtained. In the groups that Stachys lavandulifolia Vahl extracts were used for one month we observed mild degeneration of renal tubular epithelial cells (6, 9). In the second month of the study these histologic lesions significantly increased (p< 0.05). We concluded that hydroalcoholic extract of Stachys lavandulifolia has renal tubular toxicity which might continue following drug discontinuation (6, 9, 15).

Therefore, although antioxidant supplements generally have beneficial effects, as a caution it is advised to only consume such supplements under medical supervision in order to avoid any potential negative effects.
